# CD9 Tetraspanin Interacts with CD36 on the Surface of Macrophages: A Possible Regulatory Influence on Uptake of Oxidized Low Density Lipoprotein

**DOI:** 10.1371/journal.pone.0029092

**Published:** 2011-12-21

**Authors:** Wenxin Huang, Maria Febbraio, Roy L. Silverstein

**Affiliations:** 1 Department of Cell Biology, Lerner Research Institute, Cleveland Clinic, Cleveland, Ohio, United States of America; 2 Department of Molecular Cardiology, Lerner Research Institute, Cleveland, Ohio, United States of America; 3 Department of Molecular Medicine, Cleveland Clinic Lerner College of Medicine of Case, Western Reserve University, Cleveland, Ohio, United States of America; Universität Würzburg, Germany

## Abstract

CD36 is a type 2 scavenger receptor with multiple functions. CD36 binding to oxidized LDL triggers signaling cascades that are required for macrophage foam cell formation, but the mechanisms by which CD36 signals remain incompletely understood. Mass spectrometry analysis of anti-CD36 immuno-precipitates from macrophages identified the tetraspanin CD9 as a CD36 interacting protein. Western blot showed that CD9 was precipitated from mouse macrophages by anti-CD36 monoclonal antibody and CD36 was likewise precipitated by anti-CD9, confirming the mass spectrometry results. Macrophages from *cd36* null mice were used to demonstrate specificity. Membrane associations of the two proteins on intact cells was analyzed by confocal immunofluorescence microscopy and by a novel cross linking assay that detects proteins in close proximity (<40 nm). Functional significance was determined by assessing lipid accumulation, foam cell formation and JNK activation in *wt*, *cd9* null and *cd36* null macrophages exposed to oxLDL. OxLDL uptake, lipid accumulation, foam cell formation, and JNK phosphorylation were partially impaired in *cd9* null macrophages. The present study demonstrates that CD9 associates with CD36 on the macrophage surface and may participate in macrophage signaling in response to oxidized LDL.

## Introduction

CD36 is a member of the Type 2 scavenger receptor family. It recognizes multiple endogenous and exogenous ligands, including proteins containing thrombospondin type 1 structural homology regions (TSR) [1], [2]; oxidized phospholipids expressed on oxidatively midified low-density lipoprotein (oxLDL), apoptotic cells, and cell-derived microparticles [3], [4], [5]; long chain fatty acids [6]; amyloid-β [7]; falicparum malaria-infected erythrocytes; and specific components of microbial cell walls [8]. CD36 is expressed on a variety of cells including platelets [9], monocytes, macrophages, dendritic cells, microvascular endothelial cells [10], adipocytes, myocytes, and certain specialized epithelial cells [11], [12]. As a widely expressed receptor with multiple ligands, CD36 is involved in a numerous biological and pathological processes including fatty acid uptake and sensing, innate immunity, inflammation, atherosclerosis, and angiogenesis [13].

Much of the function of CD36 depends on ligand-induced triggering of specific intracellular signaling cascades. For example, TSR containing proteins inhibit angiogenesis by inducing a CD36-dependent pro-apoptotic signal in microvascular endothelial cells via direct activation of Fyn, p38 MAP kinase and caspase-3 [14], as well as up-regulation of the Fas and TNFα mediated apoptotic pathways [15], [16]. On macrophages, oxLDL induces CD36-mediated recruitment and activation of Lyn and activation of Vav family guanine nucleotide exchange factors and c-Jun N-terminal kinase (JNK)-2 [17], [18], [19]. These pro-atherogenic pathways are required for internalization of oxLDL, foam cell formation, and inhibition of migration. CD36-mediated activation of platelets shares features with the macrophage pathway in that Lyn, JNK2, and Vav are all activated by CD36 in a ligand-dependent manner, providing a mechanistic link between oxidant stress, inflammation and thrombosis [20], [21], [22], [23].

The precise mechanisms of CD36-mediated cell signaling are incompletely understood. It has 2 very short intra-cytoplasmic domains and no inherent intracellular enzymatic activity, but its carboxy-terminal cytoplasmic domain has been shown to interact with intracellular signaling proteins, including src-family kinases and MAP kinase kinases [17]. Mutations or deletions in the carboxy terminal domain abolish signaling responses in transfected cells [24], [25]. Several aspects of CD36 function and signaling are known to require functional and/or physical association with other membrane receptors, including integrins and toll-like receptors (TLR) [26], [27]. For example, uptake of apoptotic cells by dendritic cells and uptake of shed photoreceptor outer segments by retinal pigment epithelial cells involve both CD36 and α_V_β_5_ integrin [28], [29]. Certain aspects of uptake and signaling by microbial cell wall glycolipids require both CD36 and TLR-2 containing complexes, and a CD36-TLR4-TLR6 pathway has been implicated in microglial responses to oxLDL and amyloid-β [30]. The structural mechanisms by which CD36 serves as a membrane co-receptor are not well understood, but may relate in part to co-localization in membrane microdomains.

The tetraspanin family of membrane proteins has recently been implicated in cell signaling via their ability to compartmentalize other membrane proteins including integrins, along with intracellular signaling molecules, such as small molecular weight GTP binding proteins, in plasma membrane domains [31], [32]. Tetraspanins are a widely expressed, highly conserved group of more than 30 proteins that span the plasma membrane 4 times and that contain a conserved cysteine motif in their cytoplasmic amino and carboxy terminal domains [33]. Specific tetraspanins have been shown to regulate cell adhesion, migration, activation and proliferation in inflammation, immune responses, hemostasis/thrombosis, cancer metastasis, and sperm-egg fusion. Previous studies indicated that the tetraspanin CD9 could be co-immunoprecipitated with CD36 from human platelets or endothelial cells [34], [35], but no functional significance was identified. We therefore tested the hypothesis that CD9 on macrophages would interact with CD36 and contribute to CD36-mediated functional responses. Using a combination of proteomic, immuno-localization and functional approaches we now report that macrophage CD9 associates with CD36 on the cell surface and participates in CD36-dependent uptake of oxLDL.

## Results

### Co-precipitation of macrophage CD9 and CD36 by monoclonal antibodies

**Figure 1:**
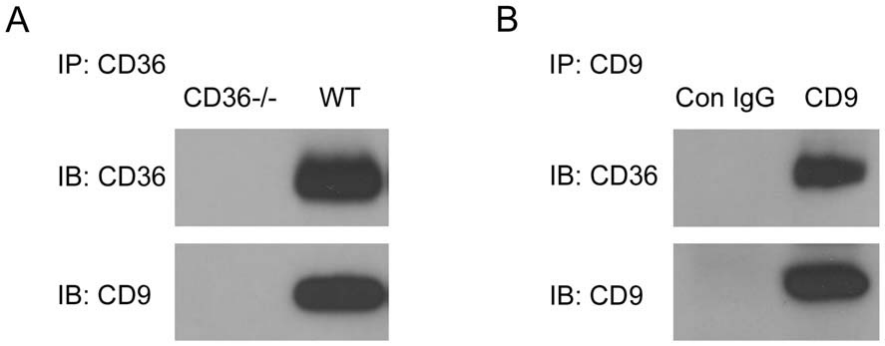
Co-immunoprecipitation of CD9 and CD36 from macrophage lysates. Peritoneal macrophages from *wt* or *cd36* null mice were lysed in 1% CHAPS and lysates containing 750 µg protein were incubated with agarose beads conjugated to murine monoclonal anti-mouse CD36 IgA (A) or anti-CD9 IgG and control rat IgG (B) at 4°C overnight. Immunoprecipitated (IP) proteins were then analyzed by immunoblot (IB) using anti-CD9 or anti-CD36 antibodies.

In preliminary experiments we used mass spectrometry to identify proteins immunoprecipitated from mouse peritoneal macrophage lysates by a monoclonal anti-CD36 IgA. The precipitates were analyzed by SDS-PAGE and then subjected to LC-MS. Multiple CD36 peptides were detected in the appropriate MW region in the gels and in the lowest molecular weight region we identified four specific peptides representing 21% amino acid coverage of CD9. CD9 peptides were not detected in immunoprecipitates from *cd36* null macrophages, demonstrating specificity. To confirm and validate these results we performed specific IPs followed by immunoblot assays. As shown in Figure 1, CD9 was detected in the anti-CD36 IP from *wt* but not *cd36* null cells (Panel A). Similarly, CD36 was detected in the anti-CD9 IP from *wt* cells (Panel B). Isotype matched control antibodies were used as controls in all studies. To further demonstrate specificity, we performed an IP with an antibody to an irrelevant macrophage surface protein, CD31, and found no evidence by western blot of co-precipitated CD36. Similarly anti-CD36 IPs did not contain detectable CD31 (not shown).

### CD9 and CD36 co-localize on the macrophage cell surface

**Figure 2:**
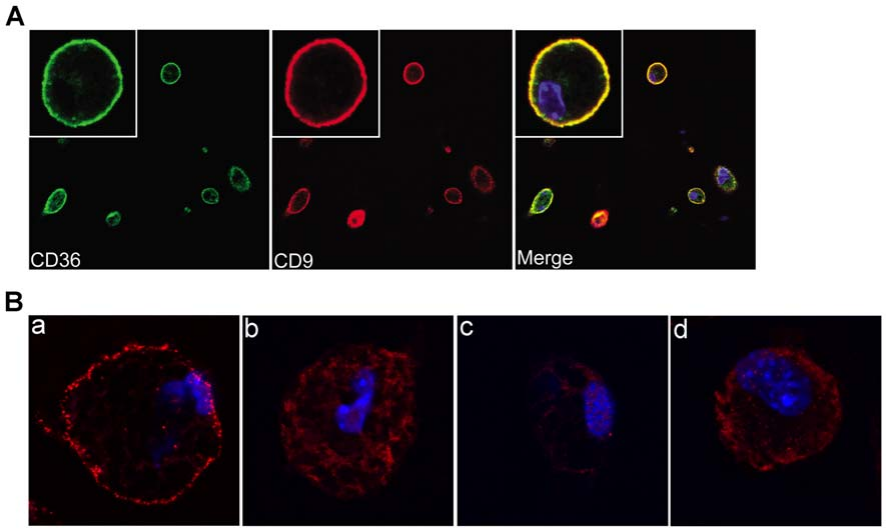
Co-localization of CD9 and CD36 on macrophage plasma membrane. *(A) Confocal Microscopy*. Mouse peritoneal macrophages were seeded on glass coverslips, fixed in 4% formaldehyde, and then incubated with FITC-conjugated anti-CD36 IgA (left panel; green fluorescence) or unlabeled rabbit anti-CD9 IgG followed by Alexa-594 conjugated goat anti-rabbit IgG (middle panel; red fluorescence). Cells were also incubated with DAPI (Blue) to detect nuclei. Confocal images were obtained at (63×); insets show (6×63×). Right panel shows merged images. (B) *Proximity Ligation Cross-linking Assay*. Macrophages from *wild type* (a) or *cd36* null (b) mice were incubated with rat anti-CD36 monoclonal IgG and rabbit anti-CD9 antibody and then species specific DNA-conjugated secondary antibodies. Specific oligonucleotides were then added, ligated and amplified using complementary fluorescent probes. Fluorescent dots represent cross-linked antibodies. In panels c and d, wild type macrophages were incubated with rabbit anti-CD36 IgG, but with anti-CD31 (c) or anti-CD40 (d) as negative controls.

Because of potential artifacts introduced by detergent lysis of membrane proteins, we also examined CD9 and CD36 association by immunofluorescence microscopy. The confocal images shown in Figure 2A demonstrate that both CD9 and CD36 are densely expressed on the macrophage cell plasma membrane in a “ring” pattern. The merged image shown in the far right panel shows nearly complete overlap of fluorescence from the two markers. We then used a Proximity Ligation Cross Linking Assay (OLink, Inc) with anti-CD9 and anti-CD36 antibodies derived from 2 different species (rabbit and mouse). In this system, species specific secondary antibodies conjugated to unique DNA strands that template hybridization of specific oligonucleotides are then added, and when in close proximity (<40 nm) the oligonucleotides can be ligated to form a circular template. The template can then be amplified and detected using specific complementary oligonucleotide probes tagged with fluorescent probes. Single-molecule protein-protein interaction events are visualized as distinct fluorescent spots. Figure 2B, panel a shows distinct spot formation in WT macrophages using this system with anti-CD9 and anti-CD36 antibodies. To show specificity we demonstrated that no spots were formed on *cd36* null cells with these antibodies (Figure 2B, panel b) and that no spots were formed when CD31 or CD40 antibodies were used instead of CD9 on WT cells (panels c and d). To confirm these results, we also used FITC-labeled anti-CD36 and biotin-labeled anti-CD9 mouse antibodies or biotin-labeled anti-CD31 rat IgG as primary antibody sets to repeat the experiment with secondary anti-FITC and anti-biotin antibodies for detection. The results were similar (not show). These studies thus show that CD9 and CD36 are in close proximity to each other (within 40 nM) on the surface of macrophages.

### CD9 participates in CD36-mediated macrophage functions

**Figure 3:**
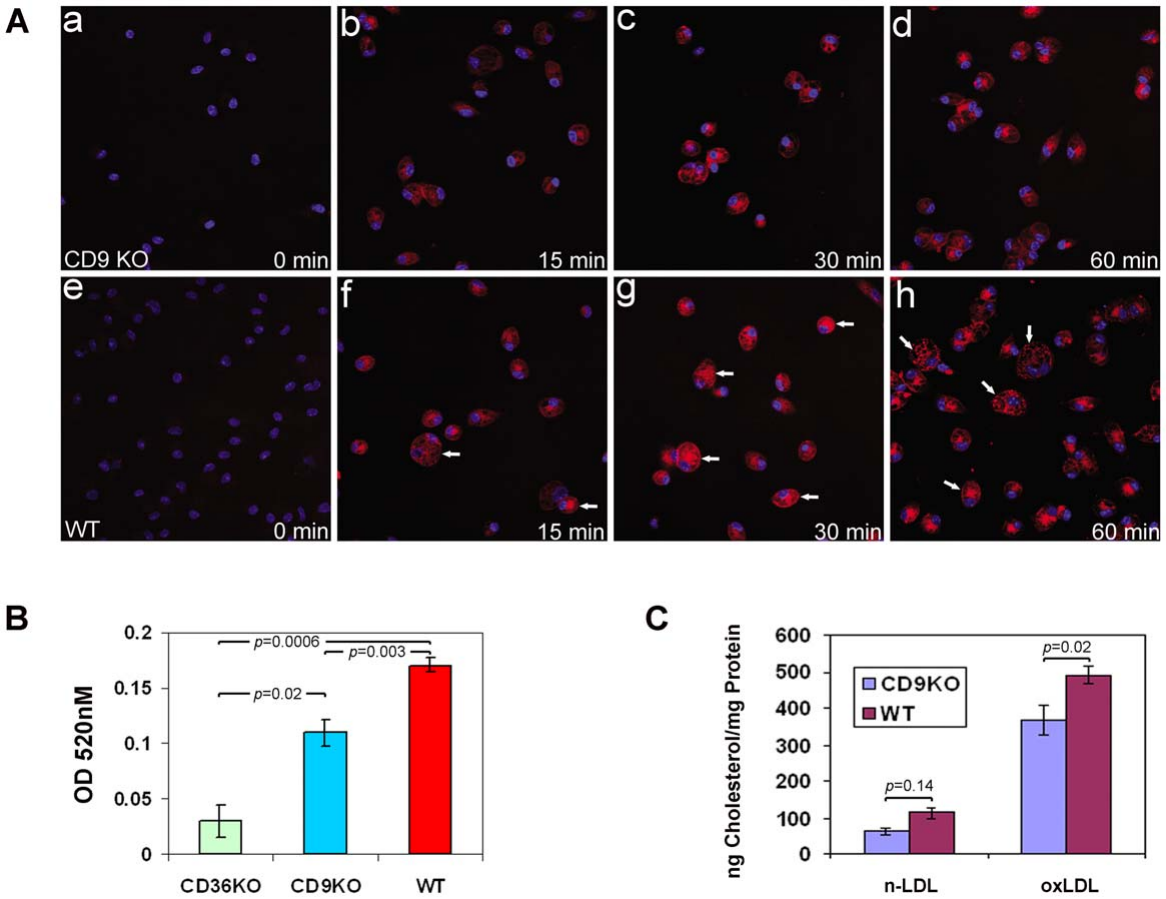
CD9 null macrophages have impaired uptake of oxidized LDL. (A) Peritoneal macrophages from *cd9* null (top row) or *wild type* (bottom row) mice were incubated with DiI-labeled NO_2_LDL (10 µg/ml) for timed periods from 0–60 minutes at 37°C. Fluorescent images show that *wild type* cells took up more DiI (red) than *cd9* nulls. Blue fluorescence represents DAPI-stained cell nuclei. White arrows indicate the macrophages with high number of lipid droplet formation. (B) Peritoneal macrophages from *cd9* null, *cd*36 null, or *wild type* mice were incubated with NO_2_LDL (50 µg/ml) for 16 hours at 37°C and then fixed and stained with oil red O for 30 minutes. After washing, the dye was extracted from the cells by methanol and detected by absorbance at 520 nM. Each group represents the mean of 3 individual samples. (C) Macrophages were treated with oxLDL as in B, or with native LDL, and then lysed. Total cellular cholesterol and protein were then quantified. Each group represents the mean of 3 individual samples.

To investigate the role of CD9 in the biological functions of CD36, we first studied oxLDL uptake and foam cell formation using macrophages obtained from *cd9* null mice. For these studies we used a form of oxidized LDL highly specific for CD36 (termed NO_2_LDL) that is generated by incubating human LDL with a myeloperoxidase/nitrite-based oxidizing system. In a short term experiment using DiI-labeled NO_2_LDL we found that fluorescence uptake at 15–60 minutes was moderately decreased in *cd9* deleted macrophage compared to *wt* macrophages (Figure 3A). To determine the quantitative impact of this defect on foam cell formation we incubated *wt* and *cd9* null macrophages with NO_2_LDL for 16 hours. Cells were then stained with Oil Red O and neutral lipid content was assessed by extracting and quantifying the dye. Figure 3B shows, as expected, that *cd36* null macrophages accumulated little or no lipid, and that the *cd9* null cells accumulated significantly less that *wt*, but more than the *cd36* null. To confirm these results we also assayed total cholesterol content in the cell lysates (Figure 3C) and showed a 26% decrease in *cd9* null cells compared to wt cells (p = 0.02). No differences among the genotypes were seen in cells incubated with native LDL. Flow cytometry assays with monoclonal anti-CD9 IgG showed that the level of surface expression of CD9 was not changed in *cd36* null macrophages (data not shown; p = 0.8).

**Figure 4:**
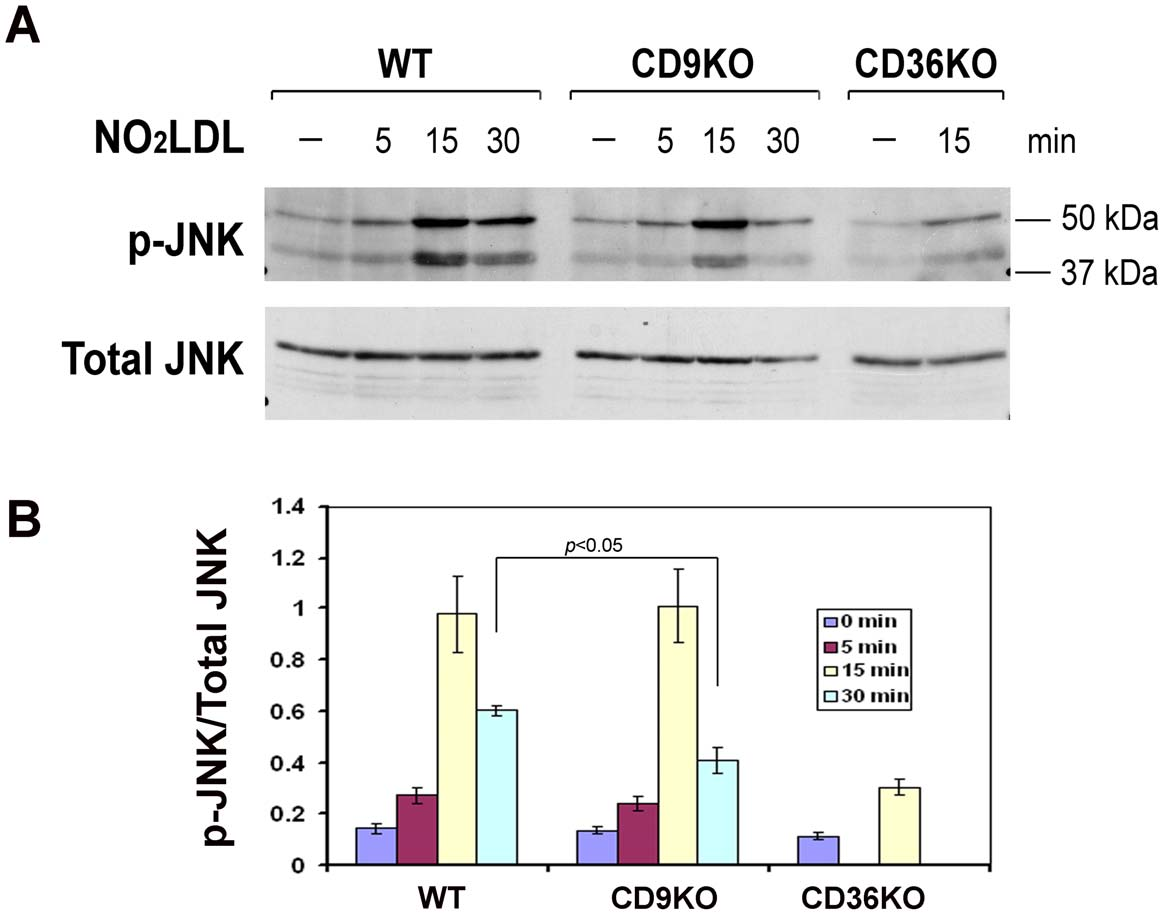
OxLDL induced JNK phosphorylation is reduced in cd9 null macrophages. (A) Peritoneal macrophages from *wild type*, *cd9* null and *cd36* null mice were stimulated by oxidized LDL (50 µg/ml) for timed points from 0–30 minutes. Cells were then lysed and analyzed by western blot with antibodies to phosphor-JNK (top) or total JNK (bottom). (B) Blots from (A) were scanned and band densities quantified using NIH Image-J software. The ratios of p-JNK/total JNK are indicated, each group represents the mean of 3 individual samples.

Previous studies from our lab revealed that phosphorylation of the MAP kinase JNK is a proximal event in CD36 signaling in macrophages and that JNK inhibition blocks CD36-mediated uptake of oxidized LDL [17]. We thus tested the hypothesis that CD9 contributes to CD36 signaling by examining the extent and kinetics of JNK phosphorylation in *cd9* null macrophages after exposure to NO_2_LDL. Figure 4 shows western blots with an antibody specific to phospho-JNK. Both JNK1 and JNK2 were phosphorylated in *wt* cells, with an approximate 6 fold increase seen at 15 minutes. Phosphorylation was still increased by more than 4 fold at 30 minutes. Interestingly, in the *cd9* null cells NO_2_LDL incubation induced a similar degree of JNK activation as in *wt* at 15 minutes, but by 30 minutes there was significantly less activation in the *cd9* cells, suggesting that CD9 might regulate this pathway. As expected, minimal JNK phosphorylation was seen in *cd36* null cells.

## Discussion

The tetraspanin CD9 (Tspan 29) is expressed on platelets, macrophages, vascular endothelial and smooth muscle cells, neuronal cells, fibroblasts, oocytes and some epithelial cells [33]. It is among the best studied of the tetraspanins and has been shown to regulate several biologically important cellular functions, including sperm-egg fusion [36], and adhesion, proliferation, and migration of nucleated cells. It is densely expressed on platelets where it appears to play a role in modulating and stabilizing aggregration. The mechanisms by which CD9 and other tetraspanins regulate cell functions remain incompletely understood, but the prevailing model is that they associate with one another and with other membrane proteins to form a “tetraspanin web” that clusters specific membrane components and intracellular signaling molecules into microdomains that facilitate signal transduction [31]. Interaction of CD9 with specific ß1 and ß3 integrins has been shown to regulate fertilization [37], migration, adhesion and platelet aggregation. In addition to integrins, CD9 also associates with the Ig superfamily adhesion molecule ICAM, and with membrane associated growth factors.

Although Maio et al. previously showed that CD9 could be co-immunoprecipitated with CD36 in human platelet lysates [34], and Kazerounian et al recently reported the association of CD36 with the tetraspanins CD9 and CD151 in endothelial cells [35], a functional role for the interaction was not shown, nor was it shown if CD9 and CD36 co-localize in intact cells. In this report we show with several different experimental approaches that CD9 and CD36 co-associate on macrophage cell membranes. Immunoprecipation with monoclonal antibodies to either protein precipitated the other, and immunofluorescence microscopy using a novel “proximity ligation cross-linking assay” demonstrated that the two proteins are closely associated (within 40 nM) with one another on the surface of the cells. Most protein interactions involving tetraspanins are not due to direct binding between specific peptide domains, with the exception that the second extracellular domain of CD9 has been shown to bind directly to integrins [33]. Whether CD9 and CD36 bind to each other directly remains to be determined.

Our studies also suggest that the CD36 signaling pathways triggered by oxLDL which lead to cholesterol accumulation and foam cell formation may be facilitated in part by its association in tetraspanin webs. Genetic deletion of CD9 did not abolish foam cell formation, but oxLDL uptake was modestly decreased as were total lipid and cholesterol accumulation. Interestingly, in the absence of CD9, CD36-mediated activation of JNK was altered, with more rapid loss of phosphorylation and thus presumably more rapid termination of the signal. JNK activation is a critical step in foam cell formation and atherogenesis, as inhibition or deletion of JNK has been shown by our group to block CD36-mediated oxLDL uptake [17] and by others to inhibit atherosclerosis in an *apoe* null mouse model [38]. We also showed that JNK inhibition in platelets blocked CD36-mediated pro-thrombotic responses [21]. Thus modulation of the dynamics of CD36-mediated JNK activation by CD9 could account for the differences seen in oxLDL uptake and foam cell formation in *cd9* null macrophages. The mechanisms responsible for the alteration in JNK phosphorylation kinetics in the absence of CD9 remains to be determined, but possibilities include changes in recruitment of src family and/or MAP kinases to the CD36 signaling complex or alteration of phosphatase function. Our studies showing less cellular lipid accumulation in the absence of CD9 are also consistent with reports that tetraspanins can traffic between the plasma membrane and intracellular vesicular compartments and therefore potentially regulate internalization pathways [39].

In summary, we showed that CD9 and CD36 co-associate on the macrophage surface, suggesting that CD36 may be part of the tetraspanin web. Loss of this association by genetic deletion of CD9 led to a modest but statistically significant decrement in CD36-mediated signaling in response to oxLDL and a concomitant modest decrease in lipid accumulation and foam cell formation.

## Materials and Methods

### Animals, antibodies and other reagents

*cd*36 null mice [40], and *cd9* null mice [41] were described previously. All mouse studies were approved by the Cleveland Clinic Institutional Animal Care and Use Committee (Approval ID is ARC08938). Peritoneal macrophages were obtained by lavage 4 d after injection with thioglycollate and adherent cells were maintained in culture. Cell culture reagents were purchased from Invitrogen, CA, USA. Antibodies to phosphorylated forms of JNK1/2 and to total JNK1/2 were from Cell Signaling, Beverly, MA. Unlabeled or biotin-conjuncted mouse monoclonal anti-CD9 antibody was from BD Biosciences, CA, USA. Rabbit anti-CD9 monoclonal antibody was purchased from Epitomics, CA, USA. Mouse anti-mouse CD36 IgA was prepared as previously described [28]. Rat anti-mouse CD36 IgG was a kind gift from Prof. Laura Helming (Munich, Germany) [42]. Rabbit anti-CD36 antibody was from Novus biologicals, CO, USA. Anti-CD31 and anti-CD40 for Proximity Ligation Cross-linking Assay were from BD Biosciences, CA, USA. LDL was isolated from human plasma as previously described [17] and oxidized with a myeloperoxidase based system as previously described [3]. In some experiments LDL was exposed to all elements of the system except the oxidant to create control non-oxidized LDL. All chemicals were obtained from Sigma (St. Louis, MO, USA) unless otherwise indicated.

### Co-Immunoprecipitation (Co-IP)

Mouse monoclonal anti-mouse CD36 IgA was coupled to NHS-activated agarose beads (GE life sciences, NJ, USA) according to manufacturer's instruction. Peritoneal macrophages were treated with Dithiobis-succinimidylpropionate and then lysed in 1% CHAPS in buffer made up of 50 mM Tris-HCl (pH 7.5), 150 mM NaCl, 1 mM EDTA, 1 mM EGTA, 2.5 mM sodium pyrophosphate, 1 mM β-glycerophosphate, 1 mM Na_3_VO_4_, and a broad spectrum protease inhibitor cocktail (Roche Applied Science, IN, USA). Lysates were centrifuged at 12000 g for 10 min and the supernatants containing 750 µg protein were incubated with antibody beads rotating overnight at 4°C. After extensive washing, beads were boiled in SDS-PAGE loading buffer and the bound material run on SDS-PAGE for further analysis.

### Mass Spectrometry

Lanes from SDS-PAGE gels prepared as above from *wt* and *cd*36 null macrophages were cut horizontally into 10 sections. The gel pieces were then reduced with DTT and alkylated with iodoacetamide before digestion with trypsin overnight. Peptides were then extracted from the gel slices and the extracts evaporated to <30 µl for LC-MS analysis using a Finnegan LCQ ion trap mass spectrometer system. The HPLC column was a self-packed 8 cm×75 µM internal diameter Phenomenex Jupiter C18 reverse-phase capillary chromatography column. Peptides were eluted from the column by an acetonitrile/0.05 M acetic acid gradient and introduced into the mass spectrometer on-line. The microelectrospray ion source was operated at 2.5 kV. Data were analyzed using all CID spectra collected to search NCBI databases with the search program Mascot.

### Immunoblot

For co-IP studies proteins from SDS-PAGE gels prepared as described above were transferred to PVDF membranes (BioRad, CA, USA) and probed with specific antibodies to CD36 and CD9 using a chemiluminescence based detection system (GE life sciences). In some studies the IP was done with anti-CD9 beads instead of anti-CD36. For studies of JNK activation, cells were treated with oxidized LDL (50 µg/ml) for timed periods and then washed twice in ice-cold PBS before lysis in 50 mM Tris-HCl (pH 7.5), 150 mM NaCl, 1 mM EDTA, 1 mM EGTA, 1% NP-40, 0.5% sodium deoxycholate, 2.5 mM sodium pyrophosphate, 1 mM β-glycerophosphate, 1 mM Na_3_VO_4_, and proteinase inhibitor cocktail. After centrifuging at 12000 g for 10 minutes, the cleared supernatants were run on SDS-PAGE, transferred onto PVDF membranes, and probed with antibodies to phospho-JNK using a chemiluminescence detection system. Blots were stripped and re-probed with antibodies to control proteins (β-actin or JNK) to assess loading. For quantification, blots were scanned and band densities determined using NIH Image-J software.

### Immnofluorescence microscopy

Peritoneal macrophages from *wt* mice were seeded on coverslips and cultured in RPMI 1640 medium supplied with 10% FCS. Attached cells were fixed in 4% formaldehyde and then incubated with FITC-labeled monoclonal anti-CD36 IgA (Cayman Chemical, Mi, USA) and/or unlabeled anti-CD9 antibody followed by Alexa-594 labeled Goat anti-rabbit antibody (Invitrogen, CA, USA). Cells were then counterstained with DAPI to detect nuclei and analyzed by laser confocal fluorescence microscopy.

### Proximity Ligation Cross-linking Assay

Fixed peritoneal macrophages prepared as above were incubated with rabbit anti-CD9 and rat anti-CD36 monoclonal antibodies. Coverslips were then washed and incubated with species specific secondary antibodies (Duolink®; Olink, Inc) conjugated to unique DNA strands that serve as templates for hybridization of specific oligonucleotides. The oligonucleotides were then added as per the manufacturer's protocol along with a ligase to form a circular template. The anchored template was then amplified and detected using complementary fluorescently labeled probes. Distinct spots representing single-molecule protein interaction events were visualized using a laser confocal fluorescence microscope.

### oxLDL uptake and foam cell formation

Peritoneal macrophages from *wt*, *cd9* null and *cd36* null mice adherent to coverslips were incubated with DiI-labeled NO_2_LDL (10 µg/ml) for timed points up to 60 minutes at 37°C. Cells were then fixed in 4% formaldehyde and internalized fluorescence examined by confocal microscopy. In other studies cells were cultured in 12 well plates, incubated with 50 µg/ml unlabeled NO_2_LDL for 16 hours, and then fixed with 4% formaldehyde and stained with Oil Red O to detect neutral lipids. After washing away non-bound dye, the internalized Oil Red O was extracted in methanol and quantified by absorbance at 520 nm using a 96 well plate reader (Spectra Max 190, Molecular Devices). Total cellular cholesterol content was also assessed in parallel cultures using a commercial kit (Cayman Chemical, MI, USA).

### Statistical analysis

*In vitro* assays were performed in quadruplicate cultures. All experiments were done using macrophages from at least three mice for each group. All numerical results are expressed as mean ± SEM. Statistical differences were determined by Student's *t* test.
